# Splenic vein stenting for recurrent chylous ascites in sinistral portal hypertension: a case report

**DOI:** 10.1186/s42155-021-00213-x

**Published:** 2021-03-03

**Authors:** Brian Covello, Jacob Miller, Roberto Fourzali

**Affiliations:** Department of Radiology, Aventura Hospital & Medical Center, 20900 Biscayne Blvd, Aventura, FL 33180 USA

**Keywords:** Sinistral portal hypertension, Splenic vein stenting, Splenic vein stenosis, Chylous ascites

## Abstract

**Background:**

Sinistral portal hypertension results from obstruction or stenosis of the splenic vein and is characterized by normal portal vein pressures and liver function tests. Gastrointestinal bleeding is the most common presentation and indication for treatment. Although sinistral portal hypertension-related chylous ascites is rare, several cases have described successful treatment with portal venous, rather than splenic venous, recanalization. Splenectomy is effective in the treatment of sinistral portal hypertension-related bleeding, although recent studies have evaluated splenic vein stenting and splenic arterial embolization as minimally-invasive treatment alternatives. Splenic vein stenting may be a viable option for other presentations of sinistral portal hypertension.

**Case presentation:**

A 59-year-old gentleman with a history of necrotizing gallstone pancreatitis was referred to interventional radiology for management of recurrent chylous ascites. Analysis of ascites demonstrated a triglyceride level of 1294 mg/dL. Computed tomography revealed splenic and superior mesenteric venous stricture. The patient elected to undergo minimally invasive transhepatic portal venography, which confirmed the presence of splenic vein and superior mesenteric vein stenosis. Venography of the splenic vein showed reversal of portal venous flow, multiple collaterals, and a pressure gradient of 14 mmHg. Two 10 mm × 40 mm Cordis stents were placed, which decreased the pressure gradient to 7 mmHg and resolved the portosystemic collaterals. At 6 months follow-up, the patient had no recurrent episodes of ascites.

**Conclusion:**

The current case highlights the successful treatment of sinistral portal hypertension-related intractable chylous ascites treated with transhepatic splenic vein stenting. Splenic venous stent patency rates of 92.9% at 12 months have been reported. Rebleeding rates of 7.1% for splenic vein stenting, 16% for splenectomy, and 47.8% for splenic arterial embolization have been reported in the treatment of sinistral portal hypertension-related gastrointestinal bleeding. The literature regarding splenic vein stenting for sinistral portal hypertension-related ascites is less robust. Technical and clinical success in the current case suggests that splenic vein recanalization may be a safe and viable option in other sinistral portal hypertension-related symptomatology.

Level of Evidence: Level 4, Case Report.

## Introduction

Sinistral portal hypertension (SPH) results from obstruction or stenosis of the splenic vein and is characterized by normal portal vein pressures and liver function tests (El Kininy et al. 2017). Gastrointestinal bleeding (GIB) is the most common presentation and indication for treatment (El Kininy et al. 2017). Although SPH-related chylous ascites is rare, several cases describe successful treatment with portal venous recanalization (Tsauo et al. 2016; Poo et al. 2018; Maleux et al. 2003). Splenectomy is effective in the treatment of SPH-related GIB, although recent studies have evaluated splenic vein stenting (SVS) and splenic arterial embolization (SAE) as minimally-invasive treatment alternatives (Ghelfi et al. 2016; Luo et al. 2014; Stein and Link 1999; Wei et al. 2020). Herein, a case of SPH-related recurrent chylous ascites successfully treated with SVS is described.

## Case report

IRB approval was not required by our institution for this case report. A 59-year-old gentleman with a history of necrotizing gallstone pancreatitis and atrial fibrillation was referred to interventional radiology for management of recurrent chylous ascites. The patient had gallstone pancreatitis 2 years prior to referral, which was complicated by the formation of recurrent and refractory infected pancreatic pseudocysts requiring repeat percutaneous and endoscopic drainage as well as a cyst-gastrostomy. During admission for cyst-gastrostomy, the patient developed bilateral sub-massive pulmonary embolism and left popliteal deep venous thrombosis while on warfarin, necessitating placement of an inferior vena cava filter. In addition, the patient reported a remote history of suspected GIB prophylactically treated with coil embolization of the gastroduodenal artery.

One month prior to referral, the patient developed chylous ascites requiring repeat paracentesis. Analysis of ascites demonstrated milky white fluid, with 89% neutrophils, 11% mononuclear cells, and a triglyceride level of 1294 mg/dL. CT imaging demonstrated focal splenic and superior mesenteric venous stenoses, gastric varices, splenoportal collaterals, and large abdominal ascites. After discussion with the patient, the decision was made to proceed with percutaneous transhepatic venography with potential venoplasty and stenting.

Under moderate sedation, ultrasound-guided transhepatic right portal access was obtained with a 21 gauge chiba needle. As the procedure involved a transhepatic approach, no intraprocedural heparin was given. Positioning was confirmed with injection of contrast under fluoroscopy. A 0.018″ nitinol (Nitrex) guidewire with a 5 Fr KMP catheter (Cook) was advanced into the portal venous system. Portal venogram was normal, with a pressure of 3 mmHg. Superior mesenteric venogram demonstrated stricture adjacent to the portal confluence and an elevated pressure of 9 mmHg. Progressive superior mesenteric venoplasty was performed up to 7 mm with a Armada 35 balloon (Abbott Vascular). No change in the pressure gradient of 6 mmHg was demonstrated.

**Fig. 1:**
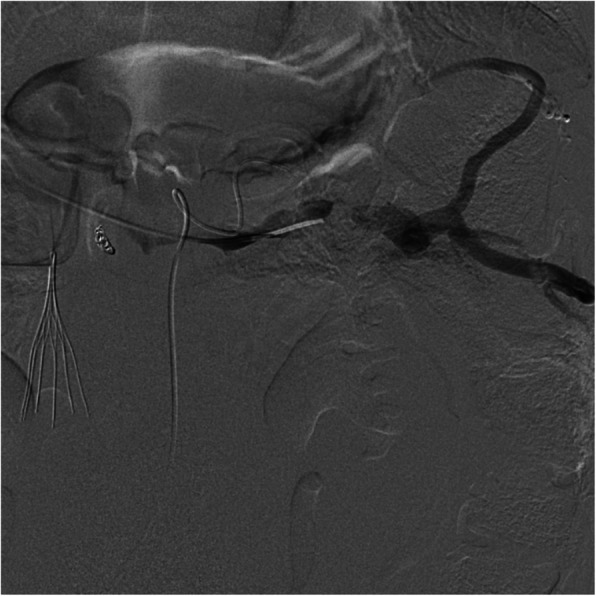
Digital subtraction venography of the splenic vein prior to venoplasty reveals multiple areas of stenosis and robust venous collaterals from portal hypertension

**Fig. 2:**
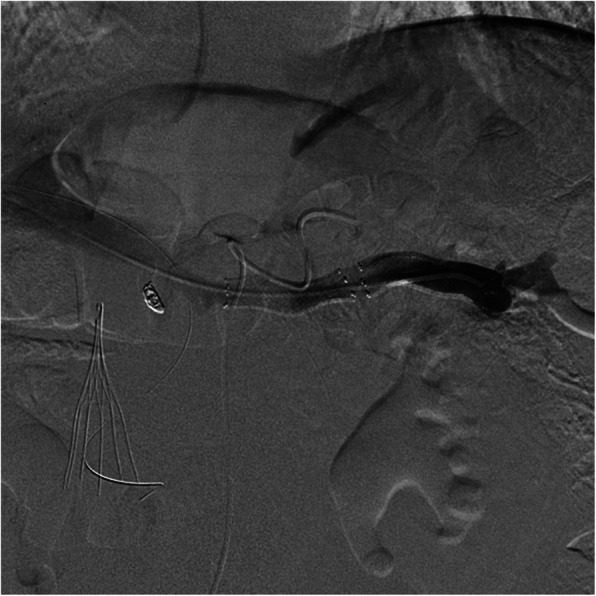
Digital subtraction venography of the splenic vein after deployment of splenic stents shows increased splenic venous patency with resolution of splenoportal collaterals

The splenic vein was then selectively catheterized. To confirm positioning within the splenic vein, the splenic artery was selectively catheterized. Digital subtraction angiogram of the splenic artery revealed delayed opacification surrounding the catheter within the splenic vein. Splenic venogram demonstrated reversal of normal portal venous flow, multiple splenoportal venous collaterals, and a splenic venous pressure of 17 mmHg (Fig. 1). Progressive splenic venoplasty was performed with a Armada 35 balloon (Abbott Vascular) up to 1 cm with no angiographic or hemodynamic improvement. Two 10 mm × 40 mm Cordis stents were placed, which resulted in resolution of splenoportal collaterals and a decrease in the pressure gradient to 7 mmHg (Fig. 2). Track embolization was performed using two 8 mm × 14 cm coils and gelfoam slurry. He was maintained on a heparin drip for 1 week before transitioning to 5 mg of apixaban twice daily. The patient developed a post-procedure perihepatic hematoma but was discharged shortly thereafter, having received no additional interventions. He remained free of ascites 6 months after the procedure.

## Discussion

Splenic vein stenosis most commonly occurs as a sequela of pancreatic disease (Luo et al. 2014; Stein and Link 1999). The patient’s history of pancreatitis with infected pseudocyst was the likely etiology of splenic venous stenosis. The most common clinical presentation of SPH is GIB, and most cases of splenic vein recanalization have treated this presentation (Fernandes et al. 2015). Several case reports have shown successful treatment of chylous ascites with portal venous stenting (PVS) (Tsauo et al. 2016; Poo et al. 2018; Maleux et al. 2003).

Splenectomy is the historical treatment of SPH-related GIB, although SVS and SAE have been advocated as possible life-saving alternatives (Wei et al. 2020; Fernandes et al. 2015). Post-splenectomy sepsis from asplenia carries a reported mortality rate as high as 6% (Loftus et al. 1993). Rebleeding rates as high as 16% have been reported (Loftus et al. 1993). SAE carries the risk of splenic abscess, post-infarction syndrome, and incomplete therapeutic response requiring splenectomy (Fernandes et al. 2015).

Luo et al. (2014) published a report on 11 patients diagnosed with SPH-related GIB who underwent transjugular splenic vein recanalization. Six of these patients were identified as having splenic vein stenosis, and technical success was achieved in each case (Luo et al. 2014). A recent retrospective comparative study between SAE and SVS for SPH-related GIB showed patients treated with SVS were less likely to develop rebleeding, with rebleeding rates of 7.1% and 47.8% in SVS versus SAE groups respectively (Wei et al. 2020). The literature regarding SVS for SPH-related ascites is less robust, with few studies describing treatment with PVS rather than SVS (Tsauo et al. 2016; Poo et al. 2018; Maleux et al. 2003; Stein and Link 1999).

SPH-related chylous ascites is caused by redistribution of gastrointestinal lymphatic flow from increased portosystemic venous pressure (Tsauo et al. 2016). In 2003, Maleux et al. first reported successful treatment of chylous ascites from portal vein stenosis with PVS, attaining resolution of chylous ascites at 1-year follow-up (Maleux et al. 2003). Stein and Link (1999) studied 21 patients with either portal vein, splenic vein, or combined portal and splenic vein stenosis. Fifteen of their patients presented with GIB, and 2 presented with intractable ascites; however, the treatment approach for patients with ascites was not specified (Stein and Link 1999). Lastly, Poo et al. (2018) reported a patient with chylous ascites who had a tight focal stenosis involving the SMV and main portal vein. The patient was treated with PVS and was ascites-free at 1-year follow-up (Poo et al. 2018). Given that the described patient presented with a non-emergent presentation of SPH, SVS was felt to be the safest option in lieu of his multiple comorbidities and preference for minimally-invasive therapy.

While no long-term patency studies of SVS for SPH-related chylous ascites are reported in the literature, comparisons may be tentatively drawn from splenic vein patency rates in SPH-related GIB. Wei et al. (2020) reported a cumulative stent patency rate of 92.9% 12 months after SVS for SPH-related GIB. A single splenic stent dysfunction was their only SVS-related complication (Wei et al. 2020). Both cases of PVS for chylous ascites in the literature reported portal venous patency at 1 year. Maleux et al. (2003) gave their patient 1 month of daily 40 mg low molecular weight heparin followed by long-term daily 160 mg acetylic salicyclic acid, while Poo et al. (2018) gave their patient long-term 75 mg daily aspirin. The patient in the current case was given 1 week of heparin drip while hospitalized, followed by his preprocedural anticoagulation of 5 mg apixaban twice daily. Anticoagulation regimen was determined based upon the patient’s history of atrial fibrillation, rather than maintenance of splenic stent patency and likely would have consisted of a less aggressive regimen in the absence of this history. There are no standardized guidelines regarding anticoagulation after portomesenteric stenting, and some studies have failed to find an association between type of anticoagulation and risk of stent thrombosis (Sheth et al. 2018). At 6 months follow-up, the patient remained free of abdominal ascites.

There has been some debate in the literature regarding a transjugular versus transhepatic approach, with some interventionalists avoiding a transhepatic approach due to the perceived risk of increased bleeding (Luo et al. 2014). Studies have shown success via both approaches, and a transhepatic approach was taken in the current case due to the relative ease of the technique and the patient’s stable bloodwork (El Kininy et al. 2017; Ghelfi et al. 2016; Luo et al. 2014; Stein and Link 1999). Nevertheless, the development of a perihepatic hematoma in the current case exemplifies the importance of weighing a variety of factors when planning an approach.

## Conclusions

This case report highlights successful treatment of SPH-related recurrent chylous ascites with transhepatic SVS. While evidence for SVS for SPH-related chylous ascites remains sparse, technical success and resolution of ascites suggest that splenic vein recanalization may be a safe and viable option. Although the current literature is promising, additional scientific studies are needed to assess the role of SVS for other SPH-related symptomatology.

## Data Availability

All data generated or analyzed during this study are included in this published article.
